# Assessing Polypoidal Choroidal Vasculopathy-Related OCT Features in the TENAYA and LUCERNE Trials

**DOI:** 10.1016/j.xops.2026.101217

**Published:** 2026-05-03

**Authors:** Kelvin Yi Chong Teo, Philippe Margaron, Timothy Y.Y. Lai, Won Ki Lee, Srinivas R. Sadda, Giovanni Staurenghi, Shriji Patel, Chui Ming Gemmy Cheung

**Affiliations:** 1 Singapore Eye Research Institute, Singapore National Eye Centre, Singapore, Singapore; 2 Duke-NUS Medical School, National University of Singapore, Singapore; 3 F. Hoffmann-La Roche Ltd, Switzerland Department of Ophthalmology and Visual Sciences, Basel, Switzerland; 4 The Chinese University of Hong Kong Hong Kong Eye Hospital, Hong Kong, China; 5 Nune Eye Hospital, Seoul, South Korea; 6 Doheny Eye Institute, David Geffen, School of Medicine, University of California- Los Angeles, Pasadena, California; 7 Department of Biomedical and Clinical Sciences “Luigi Sacco, ” Eye Clinic, University of Milan, Milan, Italy; 8 Genentech, Inc., South San Francisco, California

**Keywords:** Polypoidal Choroidal Vasculopathy (PCV), Neovascular Age-Related Macular Degeneration (nAMD), Optical coherence tomography (OCT), TENAYA, LUCERNE

## Abstract

**Objective:**

To describe OCT features associated with polypoidal choroidal vasculopathy (PCV) in the TENAYA and LUCERNE clinical trial cohorts.

**Design:**

Imaging analysis within 2 multinational, double-masked, randomized controlled phase III trials.

**Subjects:**

Participants with treatment-naïve neovascular age-related macular degeneration enrolled in TENAYA and LUCERNE with baseline OCT and in a subset, baseline indocyanine green angiography (ICGA).

**Methods:**

Previously described OCT features associated with PCV were evaluated among 1329 participants. Two major OCT features were evaluated (sharp-peaked pigment epithelial detachment [SPED] and sub-retinal pigment epithelium ring-like lesion) to determine the occurrence of OCT-defined PCV. Subretinal fluid, intraretinal fluid (IRF), and hyperreflective material (HRM), were compared between OCT-defined PCV and non-PCV eyes. A sensitivity analysis was performed in the subset of eyes with available ICGA to assess concordance between OCT and ICGA-confirmed PCV.

**Main Outcome Measures:**

Occurrence of OCT-defined PCV and distribution of OCT-based disease activity biomarkers by PCV status.

**Results:**

Using the 2 major OCT criteria, 88 (6.7%) of eyes were classified as OCT-defined PCV. Fewer OCT-defined PCV eyes had IRF and HRM compared with non-PCV eyes (27.3% vs. 45.5%, *P* = 0.002, 19.3% vs. 47.0%, *P* < 0.001). In the ICGA subset (n = 274), the combination of SPED and ring demonstrated good sensitivity (85.2%) and high specificity (98.8%) relative to ICGA.

**Conclusions:**

Using 2 Asia-Pacific Ocular Imaging Society major OCT features, OCT-based diagnostic strategy may help triage or screen for PCV when ICGA is unavailable.

**Financial Disclosure(s):**

Proprietary or commercial disclosure may be found in the Footnotes and Disclosures at the end of this article.

Polypoidal choroidal vasculopathy (PCV) is a clinically significant subtype of neovascular age-related macular degeneration (nAMD), characterized by polypoidal lesions and branching vascular networks, best visualized using indocyanine green angiography (ICGA).^1^ Although ICGA is considered the gold standard for diagnosis, its use is often limited in routine clinical settings due to accessibility, cost, and workflow challenges.^2^^,^^3^ Moreover, ICGA was not systematically included in many major phase III clinical trials, which likely resulted in underdiagnosis of PCV and an underestimation of its true occurrence and impact on treatment outcomes.^4^^,^^5^

To address this diagnostic challenge, the Asia-Pacific Ocular Imaging Society (APOIS) developed and validated a set of non-ICGA surrogates for detecting PCV.^6^ Based on a literature review and expert consensus, 9 candidate OCT and multimodal imaging features were initially proposed and their diagnostic performance evaluated. Among these, 3 features with the highest area under the receiver operating characteristic curve (AUC) were selected as major diagnostic criteria: (1) sub-retinal pigment epithelium (RPE) ring-like lesion (ring); (2) complex RPE elevation on en face OCT; and (3) sharp-peaked pigment epithelial detachment (SPED). These 3 features collectively represent the 2 key anatomical components of PCV, the polypoidal lesion and the branching neovascular network. Specifically, the ring sign and SPED reflect the polypoidal component, while the en face complex RPE elevation is associated with the branching network.

In addition to these major criteria, 4 features met the prespecified AUC requirement for inclusion as minor diagnostic criteria: the presence of an orange nodule on color fundus photography, thickened choroid with dilated Haller's layer, complex, notched or multilobular pigment epithelial detachment (PED) (mPED), and the double layer sign (DLS). Two other evaluated features, massive hemorrhage and fluid compartment involvement, did not meet the diagnostic thresholds (AUC ≤0.6) and were not pursued further. The APOIS study demonstrated that the combination of all 3 major criteria yielded excellent diagnostic performance, with an AUC of 0.90 (sensitivity 75%, specificity 91%), while combinations of any 2 major criteria also showed strong performance, with AUCs ranging from 0.82 to 0.89.^6^ The APOIS criteria were developed in several phases, which started with shortlisting of 9 individual features. The sensitivity, specificity, positive predictive value and negative predictive value, and predictive accuracy of each individual feature against ICGA as gold standard was determined based on a grading exercise by 15 panel members on 10 eyes. Major and minor criteria were selected based on prespecified AUC thresholds. Finally, the proposed diagnostic set comprising 3 major criteria had been validated in a further exercise by 6 readers from Singapore and Milan of seniority ranging from trainees to specialists on an independent image set of 110 eyes (80 Asian patients and 30 White patients).

Building on this work, the present study aimed to apply the APOIS-OCT criteria to a large, international phase III trial population from the TENAYA and LUCERNE studies.^7^ We focused on the 2 major criteria using cross-sectional OCT that are most accessible to clinicians, the ring sign and SPED as hallmark markers of the polypoidal component of PCV. Presence of these 2 features have been reported to have an AUC of 0.82, with sensitivity 0.80, specificity 0.84, positive predictive value 0.85, and negative predictive value 0.79.^6^ In addition, we also assessed the inclusion of the 2 APOIS minor features: complex or mPED and the DLS. Finally, we also examined the distribution of fluid and disease activity biomarkers—including subretinal fluid (SRF), intraretinal fluid (IRF), and hyperreflective material (HRM)—in PCV versus non-PCV eyes. As a subset of eyes in the trials underwent ICGA, an additional sensitivity analysis was performed within this subgroup to evaluate the concordance of OCT-based markers with ICGA-defined PCV.

## Methods

This study evaluated the baseline multimodal imaging of participants enrolled in the TENAYA and LUCERNE phase III clinical trials, which investigated the efficacy, durability, and safety of intravitreal faricimab in treatment-naïve patients with nAMD. Both trials were randomized, double-masked, active-comparator controlled trials. Eligibility required the presence of active macular neovascularization (MNV) choroidal neovascularization secondary to nAMD, as assessed by the reading centers, with a subfoveal component related to MNV activity confirmed by fluorescein angiography and OCT. A subset of participants underwent ICGA, which was an optional modality, allowing for the identification of polypoidal lesions and branching vascular networks when available.

This study was a post hoc analysis of deidentified imaging data derived from the TENAYA and LUCERNE clinical trials. The original studies were conducted in accordance with the Declaration of Helsinki and were approved by the relevant institutional review boards or ethics committees at each participating site. Written informed consent was obtained from all participants in the parent studies.

For this analysis, all baseline imaging including OCT, color fundus photography, and ICGA where available were submitted to the Singapore Ocular Reading Centre for centralized grading. Images were anonymized prior to submission, and the graders were masked to findings from other imaging modalities. Each image was graded by a certified grader following a standardized protocol with ambiguous or complex cases escalated to a senior retinal specialist (K.Y.C.T.) for adjudication. Each imaging modality was independently reviewed for gradable quality before feature assessment, and eyes with ungradable images were excluded from further analysis.

### Imaging Acquisition and Assessment

Anonymized spectral-domain OCT images were transmitted to Singapore Ocular Reading Centre in Digital Imaging and Communications in Medicine format. Images were acquired during the TENAYA and LUCERNE trials using Spectralis (Heidelberg Engineering, GmbH), Cirrus (Carl Zeiss Meditec), and Topcon (Topcon Medical Systems) OCT systems according to the standardized imaging acquisition protocols of the respective trials. All images were reviewed at the reading center using MicroDicom Digital Imaging and Communications in Medicine Viewer (MicroDicom Ltd, version 2024.3 [Build 3154]) for standardized assessment of OCT features.

#### OCT-Based Definition of PCV

**Figure 1:**
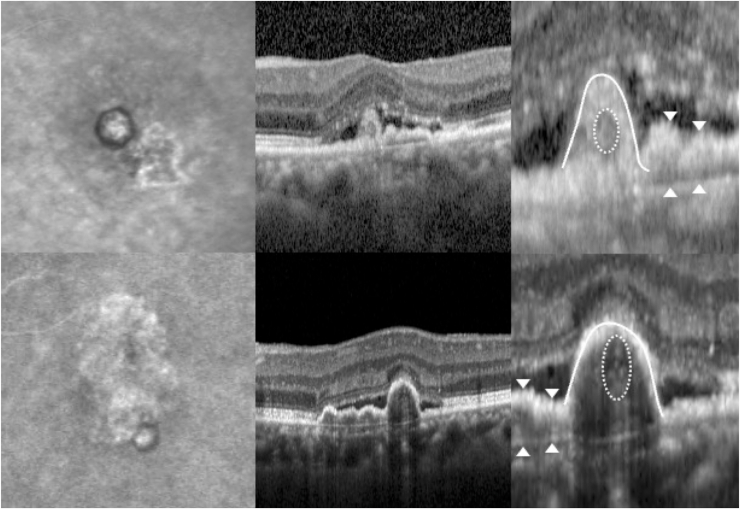
Representative multimodal imaging features used for the diagnosis of polypoidal lesions on non-ICGA imaging. Left column (top and bottom images) demonstrates polypoidal choroidal vasculopathy identified on ICGA, corresponding OCT B-scan (center column, top and bottom images), and enlarged OCT image (right column, top and bottom images). The enlarged OCT images show a sharply peaked pigment epithelial detachment outlined in white. The dashed oval outlines the ring like lesion that is suggestive of the polypoidal lesion. The double layer sign is identified between the white arrow heads. These imaging biomarkers were used as diagnostic criteria for polypoidal choroidal vasculopathy in the absence of ICGA. ICGA **=** indocyanine green angiography.

The following OCT-based markers, which have been previously associated with PCV, were evaluated. Only 2 major diagnostic criteria were evaluated: the ring sign, defined as a sub-RPE round or oval lesion with a hyperreflective border and hyporeflective or hyperreflective core (representing a polypoidal lesion in cross-section), and the SPED, defined as an inverted V-shaped PED with a steep incline (>70°) and a height-to-base ratio >1. These markers were selected based on prior validation by APOIS as reliable non-ICGA indicators of polypoidal lesions (Fig 1). The other major criterion, enface OCT, was not evaluated due to its unavailability. In addition to the major criteria, minor morphological markers were assessed as well. These included the notched or mPED, thought to represent complex or multilobulated polypoidal structures, and the DLS, a feature linked to the presence of a branching vascular network but also seen in other MNV subtypes such as type 1 MNV without polypoidal lesions (Table 1).

**Table 1:** Definitions and Diagnostic Relevance of OCT Features Associated with PCV (Based on APOIS Criteria)

Feature	Description	Likely to Represent	APOIS Criteria Level (AUC)
Ring sign	Sub-RPE round or oval lesion with a hyperreflective border and hypo- or hyper-reflective core	Polypoidal lesion	Major (0.83)
Sharp peaked PED	Inverted V-shaped PED with a steep incline (>70°) and a height-to-base ratio >1	Polypoidal lesion	Major (0.79)
Notched PED	Notch in PED, resembling an “M” OR multilobular PED	Polypoidal lesion with a large PED	Minor (0.67)
Double layer sign	Undulating RPE line that is separated from BM line underneath	Branching neovascular network	Minor (0.65)

APOIS = Asia-Pacific Ocular Imaging Society; AUC = area under the receiver operating characteristic curve; BM = basement membrane; PCV = polypoidal choroidal vasculopathy; PED = pigment epithelial detachment; RPE = retinal pigment epithelium.

#### OCT Biomarkers of Disease Activity

OCT-based markers of exudative disease activity were also graded, including SRF, IRF, and HRM. Subretinal fluid was defined as a hyporeflective space between the neurosensory retina and the RPE; IRF as hyporeflective cystoid spaces within the retinal layers; and HRM as hyperreflective signal either within or beneath the retina. These biomarkers were analyzed not as diagnostic indicators of PCV but as reflections of disease activity. In addition, subfoveal choroidal thickness was measured. This was determined as the vertical distance between the hyperreflective line of the Bruch membrane and the hyperreflective line of the inner surface of the sclera at the subfoveal position.

#### ICGA Assessment for Sensitivity Analysis

Indocyanine green angiography, when available, were graded using the EVEREST II study criteria to confirm PCV diagnosis. This required the presence of early nodular hyperfluorescence corresponding to a polypoidal lesion, accompanied by a branching vascular network. A sensitivity analysis was performed in the subset of eyes with available ICGA imaging to evaluate the concordance between OCT-defined PCV and ICGA-confirmed PCV in this cohort.

### Statistical Analysis

The primary analysis evaluated the occurrence of OCT-defined PCV within the cohort of eyes with gradable OCT imaging. Descriptive statistics were used to summarize imaging availability and the occurrences of OCT features across the cohort. Continuous variables were reported as means and standard deviations, while categorical variables were expressed as counts and percentages.

The occurrence of OCT-defined PCV was calculated as a proportion of the OCT gradable cohort. Corresponding 95% confidence intervals (CIs) were estimated using the Wilson score method for binomial proportions to provide robust estimation of uncertainty around the observed population. Comparative analyses of individual disease activity features between groups were performed using chi-square or Fisher exact tests, as appropriate. For individual fluid features (SRF, IRF, and HRM), univariate logistic regression was used to estimate crude odds ratios (ORs) with 95% CI. To account for the presence of multiple disease activity features within each eye, multivariable logistic regression analysis was performed including SRF, IRF, and HRM as covariates. Adjusted ORs with 95% CI were reported to assess the independent association of each feature with OCT-defined PCV. Disease activity feature combinations (SRF/IRF/HRM) were analyzed descriptively as mutually exclusive patterns and compared between groups using chi-square or Fisher exact tests. These combinations were not included in multivariable modeling due to collinearity with their component variables. Subfoveal choroidal thickness was compared between groups using independent sample *t*-tests.

To assess the concordance between OCT-defined PCV and ICGA confirmed PCV, diagnostic performance metrics including sensitivity, specificity, positive predictive value, negative predictive value, and AUC were calculated. Wilson 95% CIs were calculated for all diagnostic performance estimates. All statistical analyses were conducted using R version 4.3.3 (R Foundation for Statistical Computing), and statistical significance was set at *P* < 0.05.

## Results

**Figure 2:**
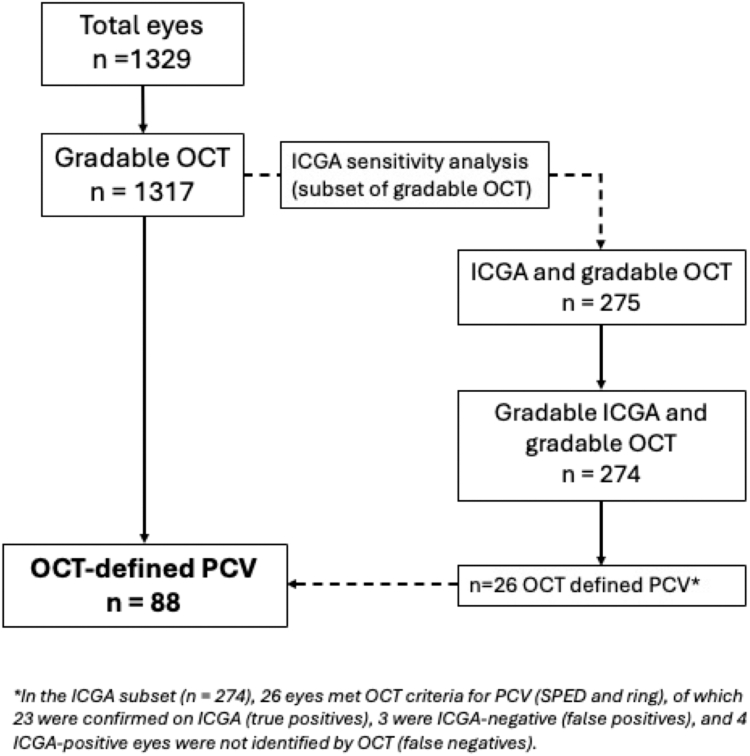
Study flow diagram and derivation of the OCT-defined PCV cohort. Flowchart showing participant inclusion, image gradability, and identification of eyes with OCT-defined polypoidal choroidal vasculopathy (PCV). Among 1329 eyes enrolled, 1317 had gradable OCT imaging. A subset of 275 eyes had both ICGA and gradable OCT available for sensitivity analysis, of which 274 were gradable on both modalities. Within the ICGA subset, 26 eyes met OCT criteria for PCV (sharp-peaked pigment epithelial detachment [SPED] and ring sign), including 23 true positives, 3 false positives, and 4 false negatives relative to ICGA. ICGA **=** indocyanine green angiography.

Images from a total of 1329 patients were submitted for analysis with OCT imaging available for all 1329 eyes, with 1317 (99.1%) deemed gradable. Indocyanine green angiography was available in 275 eyes, of which 274 (99.6%) were gradable and included in the ICGA sensitivity analysis (Fig 2).

### Occurrence of OCT-Defined PCV

**Figure 3:**
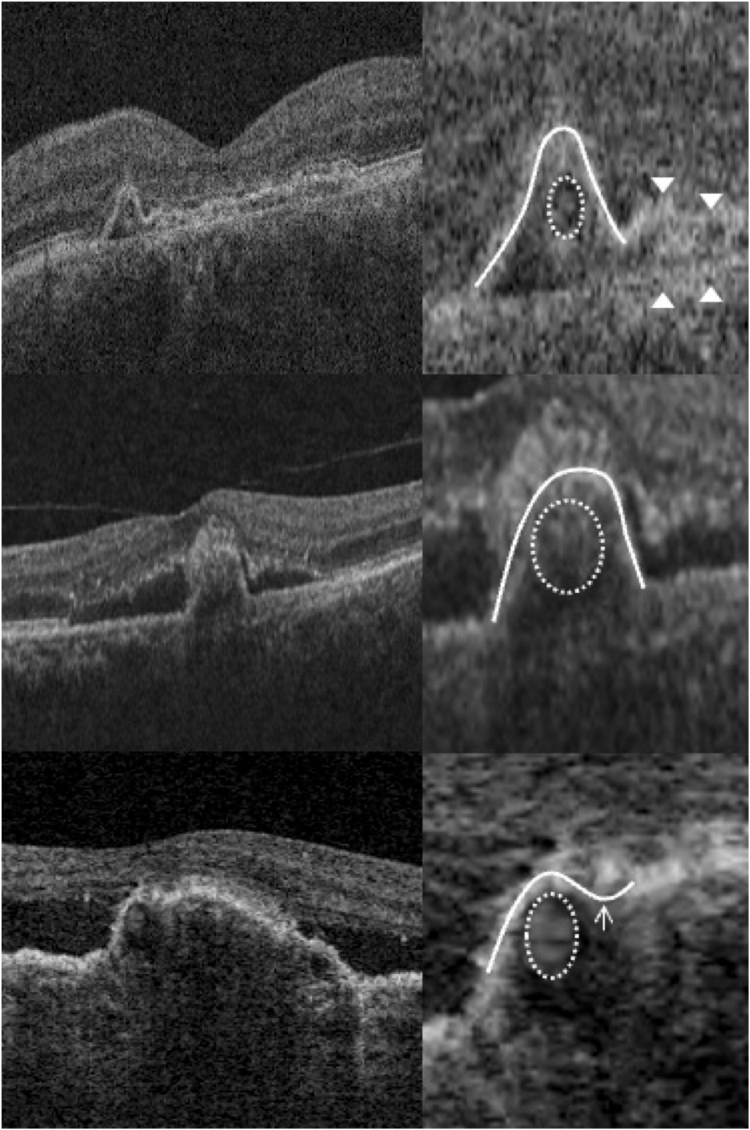
Major and minor OCT features identified in eyes with OCT-defined PCV. Representative OCT images illustrating the major and other minor OCT features used to define PCV. The right column shows magnified views of the corresponding lesions in the left column. Top row: SPED (solid white outline), ring sign (dotted circle), and adjacent DLS (arrowheads). Middle row: SPED with associated ring sign. Bottom row: notched pigment epithelial detachment (arrow) with associated ring sign. DLS = double-layer sign; PCV = polypoidal choroidal vasculopathy; SPED = sharp-peaked pigment epithelial detachment.

Using the predefined APOIS major criteria of SPED and ring sign, 88 of 1317 eyes with gradable OCT imaging met both criteria and were classified as OCT-defined PCV (6.7%, 95% CI 5.4–8.2). When the individual major OCT features were considered separately, SPED was identified in 108 eyes (8.2%) and the ring sign in 96 eyes (7.3%). Overall, a total of 117 eyes (8.9%) demonstrated ≥1 of the 2 major features (SPED or ring). Among eyes meeting both major OCT criteria, additional minor morphological markers were also observed. Notched or mPED was present in 31 eyes (35.2%), while the DLS was observed in 34 eyes (38.6%) (Fig 3).

### Distribution of Disease Activity Biomarkers

We compared disease activity biomarker profiles between OCT-defined PCV and non-PCV eyes. Among the 88 eyes classified as OCT-defined PCV and 1229 non-PCV eyes, SRF was more frequent in both groups, present in 78 of 88 PCV eyes (88.6%) and 1025 of 1229 non-PCV eyes (83.5%), with no statistically significant difference between groups (*P* = 0.12).

In contrast, IRF and HRM were less common in OCT-defined PCV versus non-PCV eyes. Intraretinal fluid was observed in 24 of 88 PCV eyes (27.3%) compared with 558 of 1229 non-PCV eyes (45.5%) (*P* = 0.002). Similarly, HRM was present in 17 of 88 PCV eyes (19.3%) versus 577 of 1229 non-PCV eyes (47.0%) (*P* < 0.001), representing the largest absolute difference among the fluid biomarkers assessed.

To account for the presence of multiple disease activity features within each eye, multivariable logistic regression analysis was performed. In this model, HRM was associated with a reduced likelihood of OCT-defined PCV (adjusted OR 0.30, 95% CI 0.17–0.51, *P* < 0.001), and IRF was also associated with a reduced likelihood of OCT-defined PCV (adjusted OR 0.53, 95% CI 0.30–0.94, *P* = 0.03). Subretinal fluid was not associated with PCV after adjustment for other fluid features (adjusted OR 0.98, *P* = 0.95).

Analysis of disease activity feature combinations demonstrated the SRF-based patterns across groups. The SRF-only pattern, defined as the presence of SRF without concurrent IRF or HRM, was present in 51 of 88 PCV eyes (58.0%) compared with 401 of 1229 non-PCV eyes (32.7%) (*P* < 0.01). The combination of SRF and IRF was observed in 15 of 88 PCV eyes (17.0%) and 145 of 1229 non-PCV eyes (11.8%) (*P* = 0.16). The combination of SRF and HRM was present in 10 of 88 PCV eyes (11.4%) versus 263 of 1229 non-PCV eyes (21.4%) (*P* = 0.03). The combination of SRF, IRF, and HRM was observed in 4 of 88 PCV eyes (4.6%) compared with 219 of 1229 non-PCV eyes (17.8%) (*P* < 0.01) (Table 2).

**Table 2:** Distribution of OCT Disease Activity Biomarkers and Fluid Phenotypes in OCT-Defined PCV vs. Non-PCV Eyes

Feature	PCV, n (%)	Non-PCV, n (%)	Unadjusted OR (95% CI)	*P*	Adjusted OR (95% CI)	*P*
Individual Fluid Features						
SRF	78 (88.6)	1025 (83.5)	1.55 (0.88–2.73)	0.12	0.98 (0.44–2.18)	0.95
IRF	24 (27.3)	558 (45.5)	**0.45 (0.27–0.75)**	**<0.01**	**0.53 (0.30–0.94)**	**0.03**
HRM	17 (19.3)	577 (47.0)	**0.27 (0.16–0.46)**	**<0.01**	**0.30 (0.17–0.51)**	**<0.01**
Fluid phenotypes (SRF/IRF/HRM combinations)∗						
SRF only	51 (58.0)	401 (32.7)	-	**<0.01**	-	-
SRF + IRF	15 (17.0)	145 (11.8)	-	0.16	-	-
SRF + HRM	10 (11.4)	263 (21.4)	-	**0.03**	-	-
SRF + IRF + HRM	4 (4.6)	219 (17.8)	-	**<0.01**	-	-
IRF only	6 (6.8)	108 (8.7)	-	0.36	-	-
IRF + HRM	2 (2.3)	93 (7.6)	-	0.08	-	-

CI = confidence interval; HRM = hyperreflective material; IRF = intraretinal fluid; OR = odds ratio; PCV = polypoidal choroidal vasculopathy; SRF = subretinal fluid.

Adjusted models include SRF, IRF, and HRM simultaneously. *P* values of <0.05 are denoted in bold.

∗ Analyses of fluid phenotype combinations are descriptive; no multivariable adjustment was performed. No adjustment for multiple comparisons was applied as analyses were exploratory.

OCT-defined PCV eyes also had thicker average subfoveal choroid measurement compared with non-OCT-defined PCV eyes (256.4 μm vs. 202.3 μm, *P* < 0.01).

### Sensitivity Analysis in the ICGA Subgroup

Concordance of OCT features of PCV with ICGA was performed on the subset of 274 eyes with gradable ICGA (from 275 eyes who had ICGA). To assess the potential selection bias, baseline OCT features were compared between eyes that underwent ICGA and those that did not. There were no statistically significant differences in the frequency of SRF, IRF, HRM, PED, and choroidal thickness (Table S1, available at www.ophthalmologyscience.org).

Within this subset, 27 eyes (9.8%) were graded as PCV on ICGA. Using the predefined OCT definition of PCV requiring both SPED and ring sign OCT demonstrated a sensitivity of 85.2% (95% CI: 66.3–95.8) and a specificity of 98.8% (95% CI: 96.5–99.7) relative to ICGA. The positive predictive value was 88.5% (95% CI: 69.8–97.6) and the negative predictive value was 98.4% (95% CI: 95.8–99.6).

**Figure 4:**
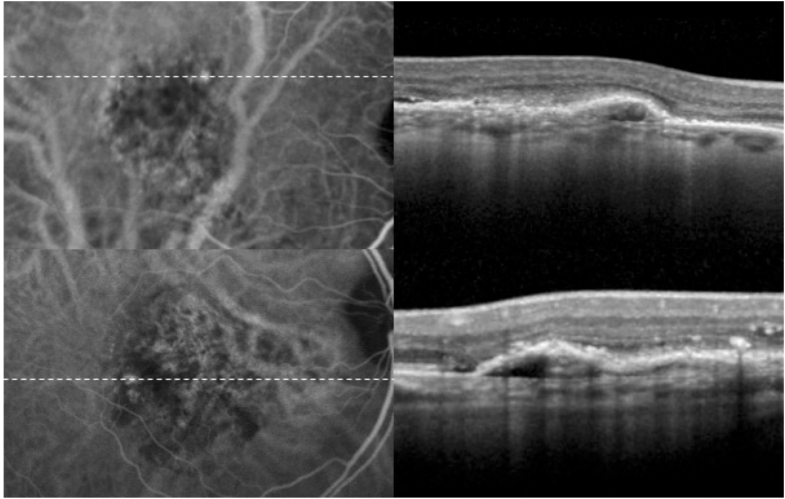
Representative examples of the OCT ring sign in ICGA-confirmed PCV. Mid-phase ICGA images (left) and corresponding OCT B-scans (right) from eyes with ICGA-confirmed polypoidal choroidal vasculopathy. The OCT ring sign appears as a hyperreflective ring surrounding a hyporeflective lumen within a pigment epithelial detachment and corresponds spatially to the polypoidal lesions identified on ICGA. Dashed lines indicate the OCT scan location. These examples demonstrate the characteristic OCT appearance underlying the high sensitivity of the ring sign for identifying PCV, including in the absence of a concomitant sharp-peaked pigment epithelial detachment (SPED), which frequently accompanies the ring sign. ICGA = indocyanine green angiography; PCV = polypoidal choroidal vasculopathy.

When considered individually, SPED alone demonstrated a sensitivity of 85.2% (95% CI: 66.3–95.8) and a specificity of 98.0% (95% CI: 95.3–99.3), positive predictive value of 82.1% (95% CI: 63.1–93.9), and negative predictive value of 98.4% (95.8–99.6). The ring sign alone showed a sensitivity of 92.6% (95% CI: 75.7–99.1) with a specificity of 96.8% (95% CI: 93.7–98.6), positive predictive value of 75.8% (57.7–88.9), and negative predictive value of 99.2% (96.9–99.9) (Table 3) (Fig 4).

**Table 3:** Concordance of OCT Features and ICGA-Confirmed PCV (ICGA Subset, n = 274)

OCT Feature	TP	TN	FP	FN	Sensitivity % (95% CI)	Specificity % (95% CI)	PPV % (95% CI)	NPV % (95% CI)
Ring sign	25	239	8	2	92.6 (75.7–99.1)	96.8 (93.7–98.6)	75.8 (57.7–88.9)	99.2 (96.9–99.9)
SPED	23	242	5	4	85.2 (66.3–95.8)	98.0 (95.3–99.3)	82.1 (63.1–93.9)	98.4 (95.8–99.6)
Ring sign and SPED	23	244	3	4	85.2 (66.3–95.8)	98.8 (96.5–99.7)	88.5 (69.8–97.6)	98.4 (95.8–99.6)

CI = confidence interval; FN = false negative; FP = false positive; ICGA = indocyanine green angiography; NPV = negative predictive value; PCV = polypoidal choroidal vasculopathy; PPV = positive predictive value; SPED = sharp-peaked pigment epithelial detachment; TN = true negative; TP = true positive.

Indocyanine green angiography served as the reference standard. Confidence intervals were calculated using the Wilson method.

## Discussion

This study evaluated several PCV-associated OCT features within the large, randomized phase III clinical trial populations of TENAYA and LUCERNE. Using 2 predefined major OCT features from the previous APOIS study, we reported approximately 6.7% of the OCT-graded cohort as having OCT-defined PCV. This figure is consistent with the reported prevalence in global clinical nAMD populations of about 5% to 10%.^1^^,^^8^, ^9^, ^10^, ^11^, ^12^, ^13^ While the original APOIS validation showed that the combination of all 3 major criteria yielded an AUC of 0.90, combinations of any 2 major criteria performed comparably well (AUC: 0.82–0.89). Specifically, the AUC of presence of SPED and ring was 0.82, with sensitivity 0.80, specificity 0.84, positive predictive value 0.85, and negative predictive value 0.79.^6^

A sensitivity analysis was performed in the subset of eyes with available ICGA to assess concordance between OCT-defined PCV and ICGA-confirmed PCV. Within this subset, the combination of SPED and ring sign demonstrated high specificity and good sensitivity relative to ICGA. These findings support the use of these OCT features as a practical approach for identifying eyes with PCV-associated characteristics when ICGA is unavailable. However, several important considerations should be noted. False-negative cases highlight both the limitations of OCT-based criteria and the heterogeneity of PCV. In our analysis, 4 ICGA-confirmed cases were not identified by SPED compared with 2 missed by the ring sign, and all false negatives in the combined criterion were driven by the absence of SPED, indicating that SPED is the principal sensitivity-limiting component. This suggests that a subset of PCV lesions may not exhibit a classic SPED configuration on OCT, instead presenting with broader or irregular PEDs, whereas the ring sign appears to capture a wider range of phenotypes consistent with its higher sensitivity.

These findings underscore the complementary roles of the 2 features: SPED enhances specificity and rule-in capability, while the ring sign improves sensitivity and detection, with their combination reflecting a trade-off between the 2. Finally, the relatively low occurrence of PCV in this cohort inflates the negative predictive value and should be considered when generalizing these results to populations with different disease subtypes.

Eyes meeting OCT-defined PCV criteria demonstrated a distinct pattern of disease activity biomarkers compared with non-PCV eyes. In particular, IRF and HRM were significantly less frequent in OCT-defined PCV, both in univariable comparisons and after adjustment for coexisting fluid features. Conversely, SRF was present more frequently in both groups and was not independently associated with PCV after multivariable adjustment. Analysis of fluid combinations further supported this pattern, with SRF-only configurations more common in OCT-defined PCV eyes, while combinations involving IRF and HRM were less frequent. Taken together, these findings suggest that OCT-defined PCV is associated with a fluid profile that has less intraretinal involvement.

These findings underscore the heterogeneity of MNV subtypes and have practical implications for clinical trial interpretation and routine management.^6^^,^^14^, ^15^, ^16^ As reporting compartment-specific fluid biomarkers is increasingly standard practice in clinical trials in nAMD, consideration should also be given to the lesion-subtype.

Key strengths of this study include the use of a large, well-characterized, international clinical trial dataset and the masked grading performed by a certified reading center. Our analysis was grounded in a widely applicable OCT-based criteria as well as a subset of eyes to analyze concordance between ICGA and OCT.

However, several limitations merit consideration. Grading was performed by a single grader per image, which precluded formal assessment of intergrader agreement. However, this was mitigated by the use of standardized grading protocols and adjudication of ambiguous or complex cases by a senior retinal specialist. Because ICGA acquisition was optional in TENAYA and LUCERNE, the validation subset may reflect site-specific practice patterns or clinical suspicion of PCV, introducing potential verification bias. Although our validation confirms strong performance of the APOIS major criteria, the absence of en face OCT complex RPE elevation in our dataset prevented assessment of the full triad of major criteria. As the TENAYA and LUCERNE trials employed strict inclusion and exclusion criteria, eyes with large neovascular lesions or central subfoveal hemorrhage were excluded, which may have led to underrepresentation of some PCV cases. Furthermore, these trials were designed to evaluate the safety and efficacy of faricimab and are not population-based cohorts. Accordingly, the frequencies reported should not be interpreted as estimates of population prevalence.

Our findings demonstrate that an OCT-based diagnostic strategy may help triage/screen when ICGA is unavailable. Incorporating OCT-based PCV identification into future trial protocols could therefore enable more precision into evaluating treatment responses in neovascular subtypes.
