# Supplementary material for: Assessing Polypoidal Choroidal Vasculopathy-Related OCT Features in the TENAYA and LUCERNE Trials
Source: Ophthalmol Sci. 2026 May 3;6(7):101217. doi: 10.1016/j.xops.2026.101217 (PMC13316682; doi:10.1016/j.xops.2026.101217)
Supplement: Table S1 [file mmc1.pdf]

Supplemental Table 1. Comparison of baseline OCT features between eyes with and without ICGA

OCT feature	ICGA (n=275)	Non-ICGA (n=1042)	p-value*
SRF, n(%)	236 (85.8%)	867 (83.2%)	0.29
IRF, n(%)	111 (40.4%)	482 (46.3%)	0.08
HRM, n(%)	115 (41.8%)	483 (46.3%)	0.18
PED, n(%)	196 (71.3%)	692 (66.4%)	0.13
Choroidal thickness, µm, mean ± SD	208.4 ± 83.4	214.5 ± 83.5	0.32

ICGA: indocyanine green angiography; SRF: subretinal fluid; IRF: intraretinal fluid; HRM: hyperreflective material, PED: Pigment epithelial detachment, SD: standard deviation

*P-values were calculated using the χ^2 test for categorical variables and the independent samples t-test for continuous variables.
